# Placental antibody transfer efficiency and maternal levels: specific for measles, coxsackievirus A16, enterovirus 71, poliomyelitis I-III and HIV-1 antibodies

**DOI:** 10.1038/srep38874

**Published:** 2016-12-09

**Authors:** Chuanxi Fu, Long Lu, Hao Wu, Jeffrey Shaman, Yimin Cao, Fang Fang, Qiongying Yang, Qing He, Zhicong Yang, Ming Wang

**Affiliations:** 1 Guangzhou Center for Disease Control and Prevention, Guangzhou, China; 2 Liwan District Maternal and Child Health Hospital, Guangzhou, China; 3 Department of Environmental Health Sciences, Mailman School of Public Health, Columbia University, New York, NY, USA; 4 College for Public Health and Social Justice, Saint Louis University, Saint Louis, USA

## Abstract

Maternal antibodies transported across the placenta can provide vital immunity against infectious pathogens for infants. We here examine maternal antibody (MA) levels and their association with neonatal antibody levels. Pregnant women of gestational age ≥35 weeks were enrolled at a Guangzhou China hospital and mother-infant paired sera were collected. Measles IgG antibody was detected using ELISA assay, neutralizing antibodies titers against coxsackievirus A16 (CA16), enterovirus 71 (EV71), PV I-III and HIV-1 were performed. 711 mother-infant pairs were enrolled and positive relationships for paired serums were found (r: 0.683–0.918). 81.6%, 87.0%, and 82.3% of mothers, and 87.3%, 72.7%, and 72.2% of newborns were positive for measles, CA16 and EV71 antibodies respectively. The highest Neonatal: maternal ratio (NMR) was found in measles (1.042) and the ratios for the other pathogens ranged from 0.84 to 1.00. Linear regressions showed that log(NMR) decreased by a factor of 0.04–15.43 as log(MA) levels increased. A second analysis restricted to maternal positive measles sera revealed that MA measles of was still inversely associated with NMR. Low NMR was found in high MA HIV + serums among 22 paired sera. MA levels appear to play a role determining transplacental antibody transfer; further study is needed to reveal the mechanism.

Maternal immunoglobulin G (IgG) is transported across the placenta by an active, neonatal Fc receptor (FcRn) mediated process during pregnancy. This transport can confer short-term passive immunity^1^,^2^,^3^ and protect infants against infections during their first months of life. Specific maternal antibodies provide immunity against infectious pathogens for infants until their own immune system has time to mature^4^.

Infectious diseases have been a threat to infants^5^. Over the past decade, measles, hand-foot-mouth disease (HFMD) and human immunodeficiency virus type 1 (HIV-1) infection have remained public health challenges among infants in some countries, including China^6^,^7^,^8^. Another disease, poliomyelitis, a crippling and potentially fatal infectious disease, may be nearing eradication by providing continued proper vaccination strategy among infants^9^.

Transplacental transport of antibodies has been shown to occur to various degrees for a variety of infectious diseases. For example, IgG transplacental transfer has been studied among preterm and term infants for certain antibodies, including tetanus, varicella, measles, and human papillomavirus (HPV). Preterm infants were found to benefit less from maternal antibodies, posing them at higher risk for infectious diseases in the first months after birth than term infants^10^,^11^,^12^,^13^. This difference may be related to the temporary decrease in total IgG during the second trimester of pregnancy due to hemodilution^14^.

Infections also influence maternal humoral immunity. Infants born to HIV-infected mothers have been found more likely to be measles antibody seronegative and had lower levels of antibodies than those born to HIV-negative mothers^15^. However, limited data are available on how maternal antibody (MA) levels influence transplacental transportation^15^. Decreasing transplacental transport of measles antibody has been reported associated with increasing levels of measles antibodies in maternal serums in some western countries and African countries^14^,^16^. However, studies of certain diseases, as well as studies in China, are lacking. Here we examine the MA levels for various antibodies of measles, HFMD, poliomyelitis virus (PV), and HIV infection, and their associations with neonatal antibody levels.

## Results

### Measles, CA16, EV71, and PV I, II, III antibodies

#### Demographic characteristics and seroprevalence of antibodies

Excluding 22 HIV + mothers, 711 mother-infant pairs were enrolled in this study with a median gestation period of 38.9 weeks (range 35–43), median delivery age of 27.7 years (range 16–45) and median birth weight of 3.2 kilograms (range 1.6–4.8). 62.7% (446) of infants were born by vaginal delivery. Antibody Levels for measles, coxsackievirus A16 (CoxA16), enterovirus 71 (EV71) and Poliomyelitis virus (PV) I, II, III in maternal and newborn serum samples are provided in Table 1. Less education (below college), diabetes and measles vaccination were related to higher maternal measles titers (p < 0.05). Less education was also linked to higher CA16 titer (p = 0.008), and women of older gestation age (≥39 weeks) were associated with higher PV II titers (p = 0.047).

Positive relationships between neonatal and maternal titers (geometric mean concentration) were found for all 7 antibodies (r: 0.918 for measles, 0.733 for CA16, 0.828 for EV71, 0.778 for PV I, 0.786 for PV II, and 0.683 for PV III; p < 0.001). 81.6% of pregnant women (95% confidence interval (CI): 78.6–84.3) and 87.3% of newborns (95%CI: 84.8–89.7) were measles antibody positive. 87.0% of pregnant women (95%CI: 82.3–90.9) and 72.7% of newborns (95%CI: 66.7–78.2) were positive for CA16 neutralizing antibodies. For EV71, the positive rates were 82.3% (95%CI: 77.0–86.8) and 72.2% (95%CI: 66.2–77.8) respectively.

#### Neonatal: maternal ratio (NMR)

The highest NMR was found for measles (1.042). The NMRs for CA16, PV I-III and HIV-1 antibodies ranged from 0.84 to 1.00. Subjects with positive maternal measles, CA16, EV71, PV I, and III antibody titers, were found to have significant lower NMRs compared with subjects with negative maternal serum levels (Table 1). Negative maternal sera also had a higher NMR for PV II antibody than the positive sera; however, the difference did not reach statistical significance. Multiple linear regressions results showed that MA titer was the common, statistically significant factor related to NMR. Indeed, NMR decreased by a factor of 0.04–15.43 as log(MA) levels increased (Table 2).

**Figure 1:**
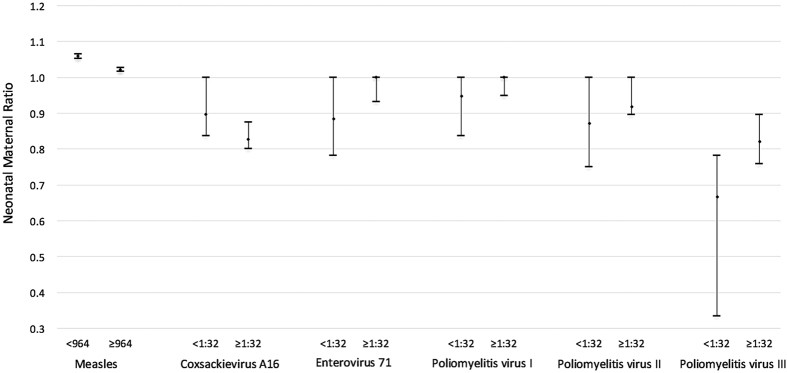
Neonatal Maternal Ratio (NMR) with 95% CIs grouped for subjects with maternal antibody levels above and below the overall median.

After we restricted the analysis to the positive maternal sera, and using the median as a cut-off, serum levels in the lower quantile showed a higher NMR than serum levels in the upper quantile for measles and CA16 (p < 0.05) (Fig. 1). The median for log (maternal antibody level) was taken as the cut-off value (2.984 for measles antibody and 1.505 for CA16, EV71, PVI, PVII and PVIII antibodies). Number of paired serums, measles: 290 (concentration <964) and 290(≥ 964); CA16: 87(<1:32) and 108(≥1:32); EV71: 84 (<1:32) and 101 (≥1:32); PV I: 87 (<1:32) and 102 (≥1:32); PVII: 75(<1:32) and 111(≥1:32); PV III:74(<1:32) and 79(≥1:32). Comparisons for NMRs between two groups: measles (t = 7.579, p < 0.001), CA16 (t = 2.436, p = 0.016), EV71 (t = −1.568, p = 0.119), PVI (t = 1.031, p = 0.304), PVII (t = −0.914, p = 0.363), and PVIII (t = −1.527, p = 0.129). For EV71 and PV I-III antibodies, maternal serum levels in the lower quantile appeared to have lower NMRs; however, the differences were not significant. In the linear regression models, NMR decreased by 0.06 as log(MA) level increased (p < 0.001) for measles, and an increase of 0.18 in NMR against log(MA) was observed for PV III antibody (p = 0.006).

#### HIV-1 neutralization antibodies

For the 22 paired mother-newborn sera for which HIV-1 neutralization antibodies were detected, the titers for 45.5% (10/22) of pairs in both mothers and newborns were 10.0. Positive relationships between neonatal and maternal titers (geometric mean concentration) were found (r: 0.819, p < 0.001). Antibody titers were found to be higher in mothers (median:19.8, 95%CI:10.0–71.9) than paired newborns (median:10.0, 95%CI: 10.0–23.2) (Z = −2.411, p = 0.016), with an average NMR of 0.957 (95%CI: 0.780–1.000). When the median of 1.2965 for log (MA titer) was used as a cutoff, the maternal sera with lower titer levels had an NMR of 1.000, and lower NMR (0.780, 95%CI: 0.627–0.913) was found in the higher titer level quantile (Mann-Whitney U = 14.000, p = 0.001).

## Discussion

Over the last decade in countries such as China, a greater number of measles, HFMD and HIV infected cases among infants have been reported^7^,^17^. At the same time new challenges arise for shifting the immunization schedule to better protect against ‘old’ infectious diseases such as poliomyelitis. MA level is an important predictor of neonatal antibody level, and maternal vaccination strategies should be recommended to provide better immunity (IgG) to neonates in the months before they are vaccinated against vaccine preventable diseases under the current national immunization schedule. For example, nearly twenty percent of pregnant women in our study were measles antibody negative. The measles vaccine with HU-191 and CHANG-47 strains has been used since 1967, and the national Expanded Program on Immunization (EPI), which used a 2-dose monovalent measles vaccine schedule, was implemented in 1986. As a consequence, maternal immunity in our study is primarily vaccine rather than naturally acquired (the oldest study subject was 45 years); however, vaccine acquired immunity may leave some women at risk to measles virus. Women of childbearing age, especially those of higher education status—a group found to have lower maternal measles titers and who are not currently advised to receive vaccination under the national schedule—should be recommended to receive a measles-containing booster, such as Measles, Mumps, and Rubella virus (MMR) vaccine, especially in the countries targeted for measles elimination in the near future^17^.

The seropositive rates of neutralizing antibody against EV71 (82.3%) and CA16 (87.0%) in women are similar with results from other areas in China (85.3% and 89.1%)^18^ but higher than other Asian and Pacific regions^19^, suggesting a higher prevalence of EV71 among the study subjects. This finding indicates that adults in China may get persistent immunity to HFMD disease by exposure to the CA16 and EV71 viruses. Because the majority of HFMD cases are reported in infants^7^, the EV71 vaccine has been licensed for use at 6–35 months.

The overall measles NMR (1.56) found here is similar to that of developed countries (>1.0) while higher than for some developing countries (<1.0)^15^,^20^. As measured by the association between cord and maternal levels, the transplacental transfer efficiency for the CA16, PV, and HIV-1 antibodies (r < 0.85) was lower than found for measles (r > 0.85). Our findings were similar to previous reporting, which showed neutralization antibody could be efficiently transferred and was strongly correlated between mothers and infants with 1.3-fold higher antibody levels in maternal plasmas^21^. More efficient transplacental transfer of measles was also reported by Goncalves^22^ for the transfer difference between measles antibody and total IgG antibodies. IgG subclasses do not cross the placenta with equal efficiency; for example, IgG1 and IgG4 are transported more efficiently than IgG3 and IgG2^2^,^3^,^10^. Such differences in IgG subclass production and accumulation may have led to the preferential transfer of measles antibody from mother to newborns. However, we did not have access to maternal IgG subclasses for this study and thus could not evaluate the associations between the IgG subclasses and NMRs for different antibodies.

We also confirm the evidence of a decrease in NMR with increasing levels of measles antibodies in maternal serum samples in China, which is consistent with findings from rural Kenya and European countries^20^,^22^. We additionally report associations between MA levels and placental transfer rates for CA16, EV71, PV I-III, and HIV-1 antibodies. The transplacental transfer efficiency for the CA16 (NMR: 0.86), EV71 (0.95), PV (0.76–1.0), and HIV-1 antibodies (0.96) was lower than found for measles (NMR:1.04). Stronger association was also found in measles antibody (r>0.9) than other antibodies (r < 0.85) in our study. It is not yet clear why MA levels for different viruses are associated with different NMRs. One hypothesis is that MA levels are associated with the total amount of IgG antibody for different viruses; IgG antibodies may compete for a finite number of FcRn receptors, which normally divert bound endocytosed IgG from catabolic degradation within the placenta by transporting them to the fetal circulation, consequently leading to different NMRs^2^,^4^,^10^,^16^. However, this mechanism was not assessed in this study. We also did not measure maternal hypergammaglobulinemia levels, which has been reported to significantly impair the transfer of IgG1 and IgG2 but not of IgG3 or IgG4^16^.

Compared with other primates, human placenta has greater active transport that concentrates IgG in the fetus during the final weeks of pregnancy, which leads to higher IgG levels in neonates than adult^23^. However, the human ability to concentrate may be impaired by gestational age, as well as infections status such as HIV and malaria infection^15^,^16^. It is possible “regression to the mean” bias, in which the observed change might depend on the initial value^24^, may have impacted this study and should be viewed as a potential limitation. Further studies of the differential concentration of antibodies as a function of age, geography, and population for various infectious diseases are needed.

To our knowledge, this is among the first studies to report the NMRs for CA16 and EV71, and to assess the associations between placental transfer rates and maternal levels for PV and HIV-1. For the 7 infectious disease antibodies examined, our study indicates that transplacental transport of antibodies occurs to various degrees and maternal antibody levels play an important role determining transfer efficiency.

## Methods

During July 2013 to April 2014, pregnant women of gestational age ≥35 weeks at Liwan District Maternal and Child Health Hospital, Guangzhou, China were randomly enrolled for this study. Maternal venous blood was sampled 2 days prior to delivery, and cord blood was obtained from the placenta directly after delivery. Sera were excluded from individuals known to be affected by an immunodepressive condition or an acute infection. Subject demographic and health information, including maternal age, education, vaccination history (measles), hypertension, diabetes, anemia, gestation week, and newborn’s gender and birth weight, was collected by study nurses.

We used an enzyme linked immunosorbent assay (ELISA; Anti-Measles IgG and IgM, Virion/Serion, Germany) for quantitative measurement of measles antibodies^25^. A modified cytopathogenic effect assay was performed to detect neutralizing antibodies titers against CoxA16/G10 (A genotpye) and EV71/H07 (C4 genotpye)^18^. In brief, sera were firstly diluted to 1:8, inactivated at 56 °C (30 minutes), then serially diluted from 1:8 to 1:2048 and mixed with equal volumes of 100 TCID50 EV71 and CoxA16. The mixture was added into a 96-well microplate and incubated at 37 °C (2 hours). Human Rhabdomyosarcoma (RD) cell suspension (10^5^ cells/ml) was added to the mixture. The plates were then placed in a CO_2_ incubator at 35 °C for 7 days, and the cytopathogenic effect was observed under microscopy. Cell control, serum control, virus control, and virus backdrops were all established on each plate. If the backdrops showed 32–320 TCID50/well, the test was considered successful. Neutralizing antibody titers were defined as the dilution rate showing 50% inhibition on the cytopathogenic effect.

PV I, II, and III antibody titers were assessed by virus-neutralizing antibody assay with Sabin or wild-type strains^26^. We also collected samples from 22 pairs of HIV + mothers and their infants in Guangzhou during 2006 to 2013. HIV-1 titer (IC50) neutralization antibody levels were determined from the curve generated by dilution and luciferase activity^21^. HIV-1 subtype B env (JRFL) plasmid and backbone plasmid pNL4.3Δenv egfp were co-transfected into 293 ft cells with 1:2 molar ratio by lipofectamine 2000. After 3 days, virus supernatant was harvested and stored at −70 °C. The virus titer was determined by blue cell assay in TZMbl cell. Plasma was 3-fold serial diluted in triplicate and incubated with 20–30 TCID50 virus per well for 1 hour in 96-well plates, then 1 × 10^4^ suspended TZMbl cells were added. After 48 h incubation, cells were lysed and luciferase activity was measured. The titer (IC50) of neutralization antibody was determined by the curve generated by dilution and luciferase activity. Equivocal assay results were retested once. Positive antibody cut-off values of 200 mIU/ml for measles, and 1:8 for CA16, EV71 and PV I-III antibodies were used in the analysis^18^,^27^,^28^,^29^,^30^.

Seroprevalence of measles, CA16, EV71, and PV I, II, III antibodies in maternal and newborn serum samples were described. NMR was defined as the geometric mean of the individual ratios, i.e. log (neonatal): log(maternal). NMRs between two groups with different maternal antibody levels were compared, in which a *t* test or nonparametric tests were used (related or dependent sample test) when appropriate. A multiple linear regression analysis was performed to determine the influence of maternal age, education, vaccination (measles), hypertension, diabetes, anemia, gestational age, newborn gender, birth weight and log(MA) on NMR. Two models were used: model 1 was analyzed based on all maternal sera, and model 2 was based on only positive maternal sera.

The sampling and experimental protocols were approved by Ethics Board of Guangzhou Center for Disease Control and Prevention (GZCDC) and all methods were performed in strict accordance with the protocol (Permit Number: 2014–007). All experimental methods were carried out in accordance with the Declaration of Helsinki. Written informed consent was obtained from the enrolled pregnant women (ClinicalTrials.gov Identifier number NCT02219061).

## Additional Information

**How to cite this article**: Fu, C. *et al*. Placental antibody transfer efficiency and maternal levels: specific for measles, coxsackievirus A16, enterovirus 71, poliomyelitis I-III and HIV-1 antibodies. *Sci. Rep.* **6**, 38874; doi: 10.1038/srep38874 (2016).

**Publisher's note:** Springer Nature remains neutral with regard to jurisdictional claims in published maps and institutional affiliations.

## Figures and Tables

**Table 1:** Seroprevalence of measles, coxsackievirus A16, enterovirus 71, and poliovirus I, II, III antibodies in maternal and newborn serum samples.

	Level	NO	Maternal serum	Neonatal serum	Neonatal Maternal Ratio^†§^
			GMT (95%CI)	GMT (95%CI)	
Measles	Maternal<200	131	78.8 (53.6, 98.6)	162.3 (113.6, 196.6)	1.128 (1.103, 1.162)
	≥200	580	963.9 (832.1, 1104.0)	1246.8 (1123.5, 1367.3)	1.036 (1.031, 1.042)
	Overall	711	674.0 (596.8, 762.0)	960.3 (819.9, 1044.1)	1.042 (1.039, 1.050)
CA16	Maternal<1:8	29	4.0^∫^	4.0^∫^	1.000
	≥1:8	195	32.0 (24.0, 32.0)	16.0 (16.0, 24.0)	0.857 (0.800, 0.896)
	Overall	224	24.0 (24.0, 32.0)	16.0 (12.0, 16.0)	0.904 (0.848, 1.000)
EV71	Maternal<1:8	39	4.0^∫^	4.0^∫^	1.000
	≥1:8	185	32.0 (24.0, 32.0)	24.0 (16.0, 32.0)	0.948 (0.911, 1.000)
	Overall	224	24.0 (16.0, 32.0)	16.0 (12.0, 24.0)	1.000 (1.000, 1.000)
PVI	Maternal<1:8	29	2.0 (2.0, 6.0)	2.0 (2.0, 6.0)	1.000 (1.000, 1.000)
	≥1:8	190	32.0 (32.0, 48.0)	32.0 (24.0, 48.0)	1.000 (0.941, 1.000)
	Overall	219	32.0 (24.0, 32.0)	24.0 (24.0, 32.0)	1.000 (1.000, 1.000)
PVII	Maternal<1:8	32	2.0 (2.0, 4.0)	2.0 (2.0, 2.0)	1.000 (1.000, 1.000)
	≥1:8	186	32.0 (32.0, 48.0)	24.0 (16.0, 32.0)	0.911 (0.884, 1.000)
	Overall	218	32.0 (24.0, 32.0)	16.0 (12.0, 24.0)	0.941 (0.896, 1.000)
PVIII	Maternal<1:8	61	2.0 (2.0, 4.0)	2.0 (2.0, 2.0)	1.000 (1.000, 1.000)
	≥1:8	153	32.0 (24.0, 32.0)	12.0 (8.0, 16.0)	0.764 (0.716, 0.837)
	Overall	214	16.0 (12.0, 24.0)	6.0 (4.0, 8.0)	0.837 (0.764, 0.911)

NOTE: The medians with the 95%CIs for CA16, EV71, PVI, PVII, and PVIII antibodies were the geometric mean concentrations.

^†^NMR = log (neonatal level): log (maternal level).

^∫^For CA16 and EV71 antibodies, all titers of maternal sera were 1:4.

^§^Comparisons for NMRs between two groups: Measles (t = 3.204, p = 0.002), CA16 (t = 5.422, p < 0.001), EV71 (t = 4.142, p < 0.001), PVI (t = 2.314, p = 0.028), PVII (t = 1.611, p = 0.117), PVIII (t = 3.467, p = 0.001).

**Table 2:** Linear models for NMRs as a function of maternal levels for different antibodies.

	Model 1^#^				Model 2*			
	NO.	Beta (95%CI)	*t*	*p*	NO.	*Beta (95%CI)*	*t*	*p*
Measles	516	−15.43 (−18.17, −12.69)	−11.05	<0.001	421	−0.06 (−0.07, −0.04)	−7.69	<0.001
CA16	208	−0.10 (−0.16, −0.04)	−3.22	0.001	180	−0.07 (−0.14, 0.02)	−1.61	0.109
EV71	208	−0.04 (−0.12, 0.03)	−1.21	0.226	171	0.06 (−0.02, 0.14)	1.49	0.138
PVI	203	−0.08 (−0.16, −0.01)	−2.11	0.037	175	0.03 (−0.06, 0.12)	0.73	0.467
PVII	202	−0.09 (−0.20, 0.01)	0.12	0.087	172	0.06 (−0.03, 0.14)	1.24	0.217
PVIII	198	−0.24 (−0.39, −0.10)	−3.30	0.001	145	0.18 (0.05, 0.31)	2.79	0.006

NOTE: The ENTER (forced Entry) method for all variables was used for the multiple linear regression models.

In both models, the dependent variable was NMR and the independent variables were maternal age, education, vaccination history (measles), hypertension, diabetes, anemia, gestation week, newborn’s gender, birth weight and Log (maternal level).

All maternal sera were analyzed in model 1 and only positive maternal sera were analyzed in model 2.

^#^In model 1, education (Beta = 0.071, p = 0.044) and birth weight (Beta = 0.123, p = 0.012) were also significant for NMR of CA16, gestation week (Beta = −2.971, p = 0.003) and maternal age (Beta = −0.014, p = 0.004) were significant for NMR of EV71, and hypertension was significant for PV II (Beta = 0.780, p = 0.005).

^*^In model 2, maternal age (Beta = −0.011, p = 0.022) for PV I and gestation week (Beta = −0.041, p = 0.031) for PVII were significant.
